# Missed Connections: Identification of Atrial Septal Defect by MRI

**DOI:** 10.1155/2023/2393308

**Published:** 2023-02-27

**Authors:** Timothy J. O'Toole, Faisal Dadi, Patrick Kietrsunthorn, Jason Foerst, Ali Hama Amin

**Affiliations:** ^1^Department of Internal Medicine, Virginia Tech - Carilion Clinic, 1906 Belleview Avenue SE, Roanoke, VA 24014, USA; ^2^Cardiovascular Institute, Virginia Tech - Carilion Clinic, 1906 Belleview Avenue SE, Roanoke, VA 24014, USA

## Abstract

In this case report, we describe a 55-year-old female patient with worsening exertional dyspnea who is referred to the cardiology department, due to the appearance of worsening pulmonary vascular disease on computed tomography (CT) of the chest. Previous transthoracic echocardiograms (TTE) identified right ventricle enlargement, but no other structural abnormalities. She completed cardiac magnetic resonance (CMR) imaging, which identified a large secundum atrial septal defect (ASD). She subsequently underwent surgical planning and correction of the lesion with improvement of her symptoms. This case and a growing body of literature support the use of CMR as an alternative imaging modality for the diagnosis of congenital heart disease (CHD).

## 1. Introduction

ASDs occur in 1–2 per 1000 births and are one of the most common congenital abnormalities in the population [1]. While these defects were typically identified in childhood, the incidence of adults with CHD is increasing due to advancements in technology and cardiac imaging [2]. ASDs will lead to the formation of a left to right shunt, which often goes unnoticed by patients as they are able to compensate for its effects and remain asymptomatic. When they can no longer compensate for the shunting, the most frequent symptoms patients will describe are fatigue, dyspnea, and exercise intolerance. Ultrasound is the current recommended modality for identifying these abnormalities. In this case report, we illustrate why CMR should be considered as an alternative modality for the identification of CHD in those patients who are unsuited for echocardiogram-based modalities.

### 1.1. Case

**Figure 1:**
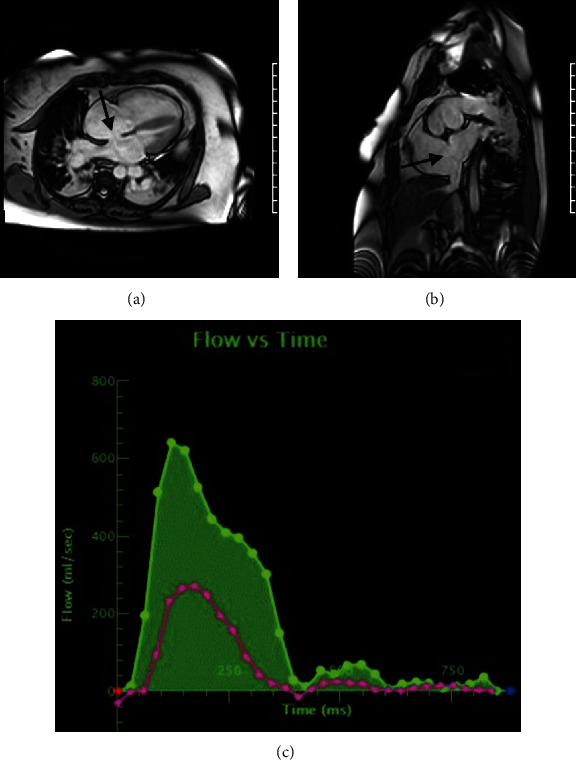
(a) CMR four-chamber view demonstrating dilated right heart with a 21 mm ASD (arrow). (b) CMR short-axis view demonstrating a 21 mm ASD (arrow). (c) CMR QP/QS of 2.9 (pulmonary flow in green and aortic flow in pink). ASD: atrial septal defect; CMR: cardiac magnetic resonance imaging; QP: right ventricular stroke volume; QS: left ventricular stroke volume.

The patient is a 55-year-old female with a history of lifelong exercise intolerance who was evaluated for dyspnea on exertion that significantly worsened over the past 3 years, now with occasional pre-syncopal episodes and inability to complete activities of daily living. Following pulmonology referral for what initially was thought to be pulmonary disease, CT of the chest without contrast was suspicious for occlusive pulmonary vascular disease. Multiple prior TTEs from outside facilities revealed right ventricle dilation, but did not demonstrate intracardiac shunts, such as ASD or patent foramen ovale, and were reported as technically difficult studies. A subsequent right heart catheterization revealed right atrial pressure 9 mmHg, right ventricular pressure 116/4 mmHg, pulmonary artery pressure 96/49 mmHg, mean pulmonary artery pressure 62 mmHg, pulmonary capillary wedge pressure 8 mmHg, and pulmonary vascular resistance 7.9 woods units. Pulmonary vasoreactivity testing with nitric oxide yielded a decrease in pulmonary artery pressure to 85/22 mmHg with a mean pulmonary artery pressure of 52 mmHg and no change in cardiac output. An oxygen saturation run demonstrated superior vena cava 67.4%, right atrium 75.7%, right ventricle 78.6%, and pulmonary artery 79.5%. The right heart pressures were most consistent with precapillary pulmonary hypertension with suspicion for group one or group four etiology; therefore, the patient was further evaluated with a V/Q scan which was negative. The partially positive pulmonary vasoreactivity test and the abnormal oxygen saturation run then raised concern for an intra-cardiac shunt. Since an ASD had not been evident on multiple prior TTEs, rarer shunts, such as a sinus venosus defect, anomalous pulmonary vein, or primum ASD, were considered; therefore, CMR was pursued for it would be more likely to identify such lesions. The patient was also started on sildenafil citrate 20 mg three times daily followed by ambrisentan 5 mg daily for pulmonary hypertension, which resulted in significant improvement in symptoms. CMR findings were consistent with a large secundum ASD with a pulmonary flow to systemic flow ratio [right ventricular stroke volume/left ventricular stroke volume (QP/QS)] of 2.9 (Figure 1).

**Figure 2:**
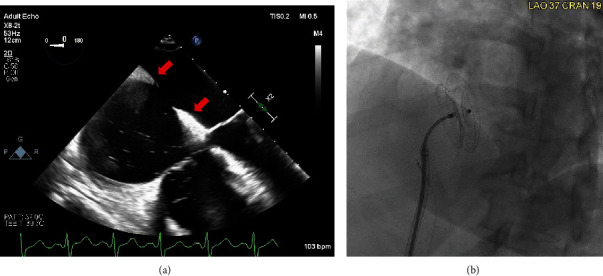
(a) TEE mid-esophageal four-chamber view confirming ASD with an adequate rim for percutaneous device closure (arrow). (b) Percutaneous deployment of a 26 mm Amplatzer Septal Occlusive device. ASD: atrial septal defect; TEE: transesophageal echocardiogram.

Following identification of this large secundum ASD, the patient was evaluated for closure of the lesion (Figure 2). An outpatient transesophageal echocardiogram (TEE) was completed for procedural planning and noted bidirectional shunting with an adequate rim for closure device. A percutaneous closure device without fenestration was placed, resolving the shunt with a resulting QP/QS of 1 and a decrease in pulmonary artery systolic pressure from 61 to 46 mmHg. TEE with bubble study completed after device deployment was negative for residual shunt. At four-week office follow up, the patient reported significant improvement in her exercise tolerance and dyspnea. At one-year follow up, the patient continued to demonstrate further improvement.

## 2. Discussion

Echocardiography is the first-line imaging modality for diagnosis of an ASD due to its availability, cost, resolution of intracardiac structures, and lack of radiation. While TTE is the preferred modality for diagnosis of the ASD due to its noninvasive nature, it can be limited by body habitus, anatomy, and operator competency. As our patient gradually became more symptomatic in the years before our evaluation, initial TTEs did not reveal any right sided chamber dilation, but did note an enlarged pulmonary artery with mildly elevated pulmonary artery mean pressure of 47 mmHg. As her symptoms continued to progress, subsequent TTEs were completed and her body mass index of 41 limited sonographers' ability to capture adequate windows. While her right atria and right ventricle were now noted to be moderately dilated, her pulmonary artery was noted to be “poorly visualized but probably normal,” and the Doppler was suboptimal for estimation of pulmonary artery pressure. This is not an uncommon problem when utilizing TTE. Additional limitations for this modality include poor windows/angles/depth (especially as patients age or undergo thoracic surgeries), suboptimal penetration, and inability to perform volumetric flow measurements [3, 4]. When TTE is equivocal, the second-line modality is TEE. This imaging modality can provide excellent spatial and temporal resolution of the heart, which is why it is favored for intraoperative use. It is the current gold standard for assessing size and rims for closure when a septal defect is present [3, 4]. The main drawbacks of TEE are its invasive nature and the need for anesthesia. The TEE guidelines established by the Society of Echocardiography found the overall complication rate to be 0.18–2.8% with the incidence of major morbidity and mortality equaling 0.2% and 0.02%, respectively [5]. The most frequently encountered complications for this procedure are lip injury (13%), hoarseness (12%), and dysphagia (1.8%), the last of which has important implications regarding aspiration and nutrition. Furthermore, the use of anesthesia carries risks of hypotension, nausea, vomiting, delirium, confusion, hypoxia, respiratory depression, and apnea [4–6]. Time to recovery and even discharge can be prolonged depending on sedatives used and patient specific factors [5]. In our case, the patient was reluctant to undergo anesthesia to complete TEE and we believed CMR would be the superior modality to identify the less common shunts mentioned above.

While echocardiography remains the first-line imaging modality for diagnosis of CHD, there is an increasing body of literature to support the use of CMR in the diagnosis, operative planning, and post-procedural monitoring of ASDs [7–9]. There is a strong correlation between defect sizes evaluated by CMR and TEE suggesting that CMR is suitable when preparing for device closure. CMR is much better suited for follow-up surveillance given its noninvasive nature when compared with TEE [8]. CMR is also superior in assessing biventricular volume and function in addition to shunt flow measurement. Unlike TEE, CMR neither carry the same invasive risks nor require anesthesia. Potential risks include the use of gadolinium contrast (which is not an absolute requirement for imaging), heating of implanted devices/foreign bodies, and heating of local tissues. Contrast can cause allergic reaction, although the incidence of this is quite small at 0.08–0.12%. The complications associated with heating of devices/implants/foreign bodies are generally mitigated by careful patient screening to identify any contraindications to CMR use [10].

In our case, the patient demonstrated life-long symptoms, which had been evaluated with multiple prior TTE exams and CT of the chest. The TTEs all demonstrated right ventricle dilation and pulmonary hypertension, but could not identify the etiology of these findings. The CT of the chest images was collected without contrast and without the specific formatting or gating required to evaluate for structural defects of the heart. The CMR identified the ASD that was not visualized on TTE or CT and identified the significantly elevated QP/QS. Due to the increasing incidence of ASDs in the adult population, a diagnosis of pulmonary hypertension should prompt a thorough investigation into the etiology to include congenital heart defects. Initial imaging should include a TTE, due to its cost effectiveness and ease of use, but if it cannot rule out a structural defect then further advanced imaging should be pursued with either CMR imaging, cardiac CT, or TEE. The decision on which imaging modality is chosen should be based on patient specific factors. Once identified, a referral to a structural cardiology center should be sought for definitive treatment and closure of the defect. Now with the increasing establishment of dedicated CMR in many facilities, this modality should be considered as an equivocal first line imaging modality in evaluation, operative planning, and post-operative surveillance of ASD.

## References

[1] van der Linde D., Konings E. E., Slager M. A. (2011). Birth prevalence of congenital heart disease worldwide: a systematic review and meta-analysis. *Journal of the American College of Cardiology*.

[2] Choe Y. H., Kang I. S., Park S. W., Lee H. J. (2001). MR imaging of congenital heart diseases in adolescents and adults. *Korean Journal of Radiology*.

[3] Babu-Narayan S. V., Giannakoulas G., Valente A. M., Li W., Gatzoulis M. A. (2016). Imaging of congenital heart disease in adults. *European Heart Journal*.

[4] Kilner P. J. (2011). Imaging congenital heart disease in adults. *The British Journal of Radiology*.

[5] Hahn R. T., Abraham T., Adams M. S. (2013). Guidelines for performing a comprehensive transesophageal echocardiographic examination: recommendations from the American Society of Echocardiography and the Society of Cardiovascular Anesthesiologists. *Journal of the American Society of Echocardiography*.

[6] Alizadehasl A., Sadeghpour A., Totonchi Z., Azarfarin R., Rahimi S., Hendiani A. (2019). Comparison of sedation between dexmedetomidine and propofol during transesophageal echocardiography: a randomized controlled trial. *Annals of Cardiac Anaesthesia*.

[7] Teo K. S., Disney P. J., Dundon B. K. (2010). Assessment of atrial septal defects in adults comparing cardiovascular magnetic resonance with transoesophageal echocardiography. *Journal of Cardiovascular Magnetic Resonance*.

[8] Weber C., Weber M., Ekinci O. (2008). Atrial septal defects type II: noninvasive evaluation of patients before implantation of an Amplatzer Septal Occluder and on follow-up by magnetic resonance imaging compared with TEE and invasive measurement. *European Radiology*.

[9] Piaw C. S., Kiam O. T., Rapaee A. (2006). Use of non-invasive phase contrast magnetic resonance imaging for estimation of atrial septal defect size and morphology: a comparison with transesophageal echo. *Cardiovascular and Interventional Radiology*.

[10] Leung S., Mukai K., Sirajudin A. (2017). https://scmr.org/page/WhyCMR.

